# Adjusted fence height: an improved phenotype for the genetic evaluation of show jumping performance in Warmblood horses

**DOI:** 10.1186/s12711-023-00786-2

**Published:** 2023-02-23

**Authors:** Léa Chapard, Anna Van Thillo, Roel Meyermans, Wim Gorssen, Nadine Buys, Steven Janssens

**Affiliations:** Department of Biosystems, Center for Animal Breeding and Genetics, KU Leuven, Kasteelpark Arenberg 30, 3001 Leuven, Belgium

## Abstract

**Background:**

Show jumping is one of the most popular disciplines in the horse sector, which makes success in show jumping competitions an important breeding goal for many studbooks. Therefore, the genetic evaluation of show jumping performance is of major interest and this is the case for two Belgian Warmblood studbooks: the Belgian Warmblood horse and Zangersheide. In this study, first an improved phenotype for show jumping performance was developed, i.e. adjusted fence height based on a new non-arbitrary method to scale ranking and competition level, which are two major components of success in competitions. Second, we assessed the importance of including a rider effect in genetic models for show jumping performance, this effect being under debate in sport horse breeding. Third, genetic models based on elementary performances and one model based on a summarized performance were compared in terms of model fit, heritabilities and the stability of estimated breeding values to define the most suitable one for the genetic evaluation of show jumping performance.

**Results:**

In this study, more than 600,000 Belgian competition records and almost 81,000 horses were used. Genetic evaluations were developed based on elementary performances (Blom-transformed ranking and adjusted fence height) and on a summarized performance (highest level achieved). Estimated heritabilities of Blom-transformed ranking, adjusted fence height and highest level achieved were 0.09, 0.12 and 0.39, respectively. Including a rider effect improved the models for genetic evaluations. Estimated genetic correlations between the studied models were moderate to high (r_g_ = 0.60–0.99). With the best fit model, the accuracy of the estimated breeding value (EBV) for adjusted fence height reached 0.70 for a larger number of stallions and for stallions that tended to be younger.

**Conclusions:**

We recommend breeders to implement this new phenotype ‘adjusted fence height’ in breeding programs. It is moderately to highly correlated with Blom-transformed ranking and highest level achieved, a proxy for lifetime success, and is available for selection candidates from an early age onwards.

## Background

The ultimate breeding goal of most Warmblood studbooks is to breed top sport horses, i.e. horses that are successful at the highest level in competitions [1]. Being successful in competitions implies being highly ranked and also participating in the most difficult competitions. Consequently, many breeding organizations use performance in competitions as the main trait in their breeding program. However, no common performance trait has ever been adopted by the different Warmblood studbooks [2]. In fact, since there is no gold standard, several breeding organizations have developed their own genetic evaluation for show jumping performance using custom phenotypes (Table 1). Some organizations use elementary performances (performances in a single competition), such as ranking in competitions, penalty points or earnings, which in some cases are transformed into more normally distributed variables [2]. Estimates of heritabilities are rather low (0.01–0.17) for these traits (Table 1). Summarized performances are an interpretation of multiple elementary performances of a horse over a year, over several years or over a lifetime. Such summarized performances show moderate heritabilities (0.31, 0.12–0.28 and 0.10–0.36, respectively) and represent the advantage of having only one observation per horse (Table 1). However, compared to a summarized phenotype, repeated measures may be more informative to estimate the genetic merit since some effects, such as the rider(s) and the other competitors in the events, can be taken into account.

**Table 1:** Review of reported heritability estimates for show jumping performance traits used in Europe

Performance trait	Countries	Heritability	References
*Elementary performances*			
Ranking (Blom transformation)	Belgium, Hungary, Ireland, Slovak Republic	0.02–0.10	[3–7]
Ranking (Normal transformation)	Hungary, Ireland	0.05–0.09	[8–10]
Ranking (Root transformations)	Hungary, Germany	0.02–0.11	[4, 8, 11, 12]
Ranking (Cotangent transformation)	Hungary	0.04	[4]
Ranking (Underlying variable)	France	0.16	[13]
Penalty points	Czech Republic	0.04	[14]
Penalty points (Logarithmic transformation)	Czech Republic	0.07	[14]
Penalty points (Blom transformation)	Czech Republic	0.07	[14]
Penalty points (Positive transformation)	Spain	0.09	[15]
Profit of penalty points (Blom transformation)	Slovak Republic	0.17	[7]
Auxiliary penalty points	Czech Republic	0.04	[14]
Auxiliary penalty points (Logarithmic transformation)	Czech Republic	0.03	[14]
Auxiliary penalty points (Blom transformation)	Czech Republic	0.05	[14]
Difference between fence height and fault points	Hungary	0.01–0.07	[4, 5]
Hungarian grading scores	Hungary	0.01–0.02	[8]
*Summarized performance*			
Over a lifetime			
Highest performance achieved (arbitrary points)	The Netherlands, Germany	0.10–0.36	[16–19]
Lifetime performance rating	Ireland	0.28	[20]
Accumulated points	Sweden	0.27–0.34	[21]
Accumulated placings	Sweden	0.26–0.30	[21]
Accumulated points per placing	Sweden	0.18–0.28	[21]
Over several years			
Accumulated points	Sweden	0.24–0.28	[21]
Placings	Sweden	0.23–0.26	[21]
Accumulated points per placing	Sweden	0.12–0.17	[21]
Over a year			
Sum of points	France	0.31	[22]

Nevertheless, defining a precise phenotype solely does not guarantee the quality of a genetic evaluation since the inclusion of other non-genetic factors can heavily affect the outcome. In the equestrian world, there is debate on the rider effect [15, 19, 23–26]. First, show jumping horses cannot perform without a rider, and second, performance in competitions does not merely rely on a horse’s capacity but also on the quality of the rider and the relationship between horse and rider. Previous studies have shown that the rider effect accounts for a significant proportion of the variance (up to 37%) in genetic evaluations for performance in competitions [15, 19, 23–25]. However, this effect can be difficult to estimate when the number of horses per rider is small or when many horses are ridden by only one rider, since it can be confounded with the quality (genetic + permanent environment effects) of the horse. The Belgian model, currently used by the Belgian Warmblood horse (BWP) studbook [3], does not consider a rider effect.

To account for both the ranking (intra-competition variability) and the competition level (inter-competition variability) i.e. to differentiate the intra- from the inter-competition variability, two strategies are used in sport horse breeding. The first one uses a rank-based phenotype [3–13] and corrects for competition level in the genetic model by adding an event effect. A rank does not reflect competition level as there will always be a winner in competitions, regardless of the difficulty. Adding an event effect allows taking competition level into account by measuring the level of the competitors, i.e. their results in other competitions. The second strategy is to develop a phenotype based on arbitrary points that depend on ranking and competition level (Table 1). The number of these assigned points differs across countries (Table 1).

Here, we initiated a study to assess simultaneously the choice of the phenotype and the importance of the rider effect in order to generate an optimal model to evaluate the genetic merit of show jumpers. To this end, we developed a non-arbitrary method to distinguish the intra- from the inter- competition variability of performances by defining a new precise show jumping performance trait based on elementary performances from competition data, i.e. “adjusted fence height” (AFH). Then, we compared this model with other models that are based on elementary performances (Blom-transformed ranking (BTR)) or on a summarized performance (highest level achieved (HL)) to define the most suitable genetic model for show jumping performance in the Belgian Warmblood studbooks.

## Methods

### Datasets

Three datasets were provided by K.B.R.S.F. (Royal Belgian Sport Equestrian Federation). The first dataset contained competition results: ranking of the horse, its sport number (a unique number given by the organization), the  fault(s), the time of the rounds, the rider identification number and the competition identification number. The second dataset included event information: the identification number, date, and location of the competition, and the fence height. The third dataset consisted of horse information: the sport number of the horse, its Universal Equine Life Number (UELN), name and sex. In total, 2,436,461 show jumping competition records, reflecting individual placings of horses within the complete ranking of each competition, from events held between 2004 and 2019, were used in this study. Occurrences of horses per rider and riders per horse are given in Table 2. An integrated pedigree [27] containing BWP and Zangersheide (Z) pedigree information such as the UELN of the horses and of their parents and the birth dates (393,719 records) was used for the genetic analyses. Among those 393,719 pedigree records, 80,897 were informative i.e. for horses with show jumping performances or relatives of horses with own performances. The following criteria were used to link the data between datasets and to clean the data using custom R scripts [28]: (i) competition records that could not be linked to an event, with impossible ranking or with missing fence height (79,426 records), (ii) records that could not be linked to a sport number (131,775 records), (iii) records of horses that could not be linked to the pedigree via the UELN due to incomplete recording of UELN or presence of foreign horses (1,379,547 records), (iv) records of horses that were younger than 4 years or older than 18 years (21,670 records), and (v) records of riders that had competed with only one horse (149,516 records) were discarded, resulting in a final number of 674,527 records retained for further analysis. These competition records referred to 50,913 unique competitions, 26,351 different horses and 8410 unique riders.

**Table 2:** Occurrences of horses per rider and riders per horse in the initial dataset (n = 2,436,461 records)

Occurence	Number of horses per rider	Number of riders per horse
1	12,128	36,064
2	5593	19,004
3	3155	10,562
4	2007	6066
≥ 5	7753	7781

### Models for analysis

First, a Blom transformation [29] was applied, which is an approximation of the “normal score” of ranks and depends on the rank and number of competitors. It was used on rankings from elementary performances resulting in Blom-transformed rankings:

$$Blom-transformed \, ranking={\Upphi}^{-1}\left(\frac{r - \frac{3}{8}}{n+ \frac{1}{4}}\right),$$

where ${\Upphi}^{-1}$ is the inverse standard cumulative normal distribution; $r$ is the rank of an observation and $n$ is the number of competitors in a competition. Blom-transformed rankings were limited to [− 2.76, + 2.76] in our data because there were, at most, 216 competitors in the same competition.

In addition to the frequently-used Blom-transformed ranking, a new phenotype “AFH” was defined using a non-arbitrary method to distinguish the intra- from the inter-competition variability of performances. For that purpose, the ranking and the competition level (defined here as fence height) were scaled by using a linear regression on the differences in fence height and the differences in Blom-transformed ranking. The differences were calculated within horses from consecutive performances in competitions (Table 3) resulting in the following regression line (r^2^ = 0.04):

**Table 3:** Descriptive statistics of differences in fence height and in Blom-transformed ranking

	n	Mean	SD	Min	Median	Max
Δ(Fence height)	674,527	0.44	7.70	− 70.00	0.00	65.00
Δ(Blom-transformed ranking)	674,527	0.00	1.17	− 4.80	0.00	4.78

$$\varDelta (Fence \, height) = -1.32 \times \varDelta (Blom{\text{-}}transformed \, ranking)+0.44.$$

We used the absolute value of the regression coefficient obtained to convert the differences in rankings, expressed as Blom-transformed rankings into the scale “fence height” in cm resulting in the following formula used to express AFH:

$$\mathrm{AFH}=\mathrm{Fence \, height}+ 1.32 \times \mathrm{Blom}\mathrm{-transformed \, ranking}.$$

AFH was then calculated for each elementary performance. In addition to the two rank-based phenotypes previously described, we constructed a summarized and career-linked trait as used in several countries and/or studbooks [16–22], i.e. the highest level achieved (HL). For each horse (n = 26,351), the highest AFH obtained during its career was extracted. Thus, in summary, three phenotypes were used in our study: two obtained from elementary performances (BTR and AFH) and one from a summarized performance (HL).

Variance components and breeding values for BTR, AFH and HL were estimated using univariate models with the remlf90 program, which is a part of the blupf90 family of programs [30]. Assessment of goodness-of-fit of the different models was based on Akaike’s information criterion (AIC) [31].

Genetic parameters for BTR, AFH and HL were estimated with the following models:

M1_BTR: $${y}_{ijkl}= \mu +{sex}_{i}+{age}_{j}+{animal}_{k}+{c}_{k}+{event}_{l}+{e}_{ijkl}$$,

M2_BTR: $${y}_{ijklm}= \mu +{sex}_{i}+{age}_{j}+{animal}_{k}+{c}_{k}+{event}_{l}{+ rider}_{m}+{e}_{ijklm}$$,

M1_AFH: $${y}_{ijk}= \mu +{sex}_{i}+{age}_{j}+{animal}_{k}+{c}_{k}+{e}_{ijk}$$,

M2_AFH: $${y}_{ijkm}= \mu +{sex}_{i}+{age}_{j}+{animal}_{k}+{c}_{k}+{rider}_{m}+{e}_{ijkm}$$,

M1_HL: $${y}_{ijk}= \mu +{sex}_{i}+{age}_{j}+{animal}_{k}+{e}_{ijk}$$,

where ${y}_{ijkl}$ is the value of the trait (BTR, AFH or HL) for the *k*th animal; $\mu$ is the population mean; ${sex}_{i}$ is the fixed effect of the sex of the horse (M, F, G); ${age}_{j}$ is the fixed effect of its age (from 4 to 18); ${animal}_{k}$ is the random additive genetic effect (from 1 to 80,897); ${c}_{k}$ is the permanent environment effect linked to animal $k$, ${event}_{l}$ is the fixed effect of the event (from 1 to 50,913); ${rider}_{m}$ is the random effect of the rider (from 1 to 8410) and ${e}_{ijklm}$, ${e}_{ijkm}$, or ${e}_{ijk}$ is the random residual effect.

The influence of the rider effect was assessed by comparing the models M1_BTR with M2_BTR and M1_AFH with M2_AFH. Genetic correlations between the five models M1_BTR, M2_BTR, M1_AFH, M2_AFH and M1_HL were estimated using bivariate analyses.

### Validation of the most suitable model

Two different groups of stallions were studied to validate the most suitable model for selection on success in show jumping competitions: (1) the approved stallions that meet the criteria for publication of their estimated breeding values (EBV), which for stallions in Belgium are currently: having an accuracy of EBV higher than 0.70 and having at least five offspring with own performances; and (2) the young stallions that were 4 to 5 years old with own performances but without offspring. Dubois et al*.* [32] showed that the optimal selection age was around 4 or 5 years old for males with own performances in competitions or in station test. The most appropriate/fitting models for BTR, AFH and HL were used to compare the two groups of stallions (1) and (2) in terms of: number of approved stallions that meet the criteria for EBV publication per model, number of young stallions that reach an EBV accuracy of at least 0.70, descriptive statistics for each group per model and Spearman rank correlations between EBV.

## Results

**Fig. 1:**
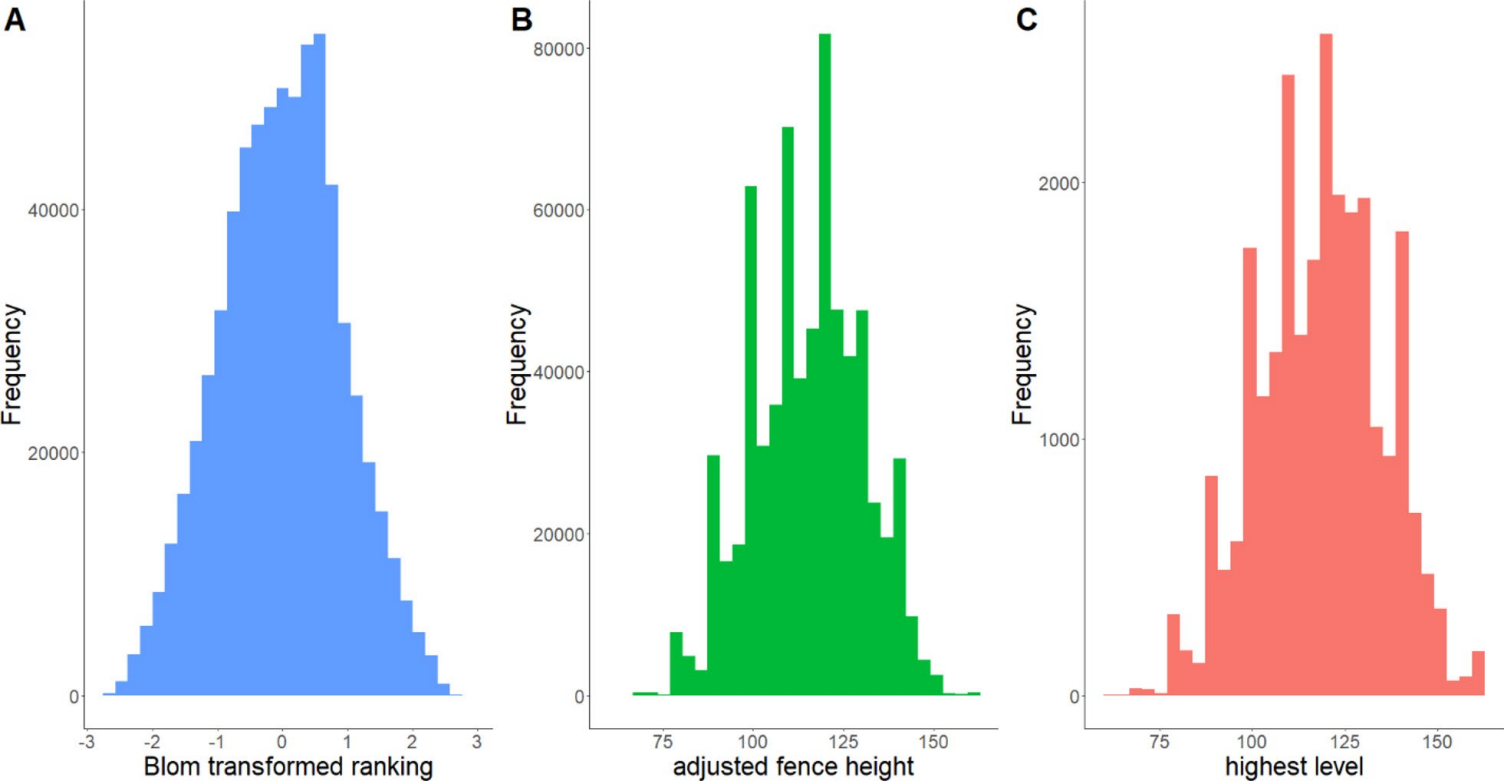
Distributions of BTR, AFH and HL in show jumping competitions: **a** Blom-transformed ranking; **b** adjusted fence height; and **c** highest level

The descriptive statistics and their distributions for BTR, AFH and HL are in Table 4 and Fig. 1. Values for BTR ranged from − 2.76 to 2.76 with a mean of 0.00 and those for AFH and HL ranged from 62.90 to 162.30 with means of 115.15 and 119.46, respectively.

**Table 4:** Descriptive statistics for BTR, AFH and HL

	n	Mean	SD	Min	Median	Max
BTR	674,527	0.00	0.94	− 2.76	0.00	2.76
AFH	674,527	115.15	15.16	62.90	115.70	162.30
HL	26,351	118.63	16.64	62.90	119.46	162.30

*BTR* Blom-transformed ranking, *AFH* adjusted fence height, *HL* highest level achieved, *n* number, *SD* standard deviation

The AIC values and estimated variance components for the different models applied to BTR, AFH and HL are in Table 5. The estimates of the heritabilities (h^2^) for BTR ranged from 0.06 to 0.09. The best-fit model for BTR was M2_BTR with an estimated heritability of 0.06. The estimates of the heritabilities for AFH ranged from 0.12 to 0.36. According to the AIC values, M2_AFH was the best-fit model. An intermediate heritability was found for HL (h^2^ = 0.39).

**Table 5:** AIC values and variance components of the models performed for BTR, AFH and HL

Model	AIC	∆AIC	Variance				Share in the variance		
			Residual	Rider	Permanent environment	Genetic	ri^2^	c^2^	h^2*^
M1_BTR	1,736,813		0.75		0.09	0.09		0.09	0.09
M2_BTR	1,726,175	− 10,638	0.74	0.07	0.08	0.06	0.08	0.08	0.06
M1_AFH	4,784,826		62.50		55.25	65.01		0.30	0.36
M2_AFH	4,605,905	− 178,921	47.03	102.10	28.46	24.35	0.51	0.14	0.12
M1_HL	211,323		119.60			76.51			0.39

∆AIC: difference in AIC between M2 and M1, ri^2^: proportion of the variance due to rider effect, c^2^: proportion of variance due to the permanent environment effect, and h^2^: heritability estimate

*Standard errors ranged from 0.004 to 0.02

When the model accounted for the rider effect (M2_BTR and M2_AFH), the AIC decreased for BTR and AFH, which means that including this effect improved the model fit, and their heritability estimate also decreased (0.09 ± 0.0051 vs 0.06 ± 0.0041 and 0.36 ± 0.0130 vs 0.12 ± 0.0061, respectively). For M2_BTR, the proportion of variance due to the rider effect was similar to that due to the permanent environment effect (0.08). Both these effects had a larger influence on M2_BTR than the additive genetic effect (0.08 ± 0.0033 > 0.06 ± 0.0041). For M2_AFH, the largest proportion of the variance was due to the rider effect (0.51): nearly four times that due to the permanent environment effect or the additive genetic effect. The permanent environment effect accounted for a slightly larger proportion of the variance than the additive genetic effect (0.14 ± 0.0048 > 0.12 ± 0.0061). Hence, both rider and environment effects had a larger influence on M2_AFH than the additive genetic effect.

The heritability estimated with the most appropriate/fitting model (M2_AFH, M1_HL, and M2_BTR) differed for the three performance traits, i.e. it was highest with M1_HL (h^2^ = 0.39), lowest with M2_BTR (h^2^ = 0.06) and low to moderate with M2_AFH (h^2^ = 0.12).

The estimated genetic correlations between M2_AFH, M1_HL and M2_BTR ranged from 0.60 to 0.99 (Table 6) and were highest between M1_AFH and M1_HL and between M2_AFH and M1_HL, and lowest between M2_AFH and M1_BTR. The estimated correlations of the permanent environment effects ranged from 0.73 between M2_BTR and M1_AFH to 0.99 between M1_BTR and M2_BTR.

**Table 6:** Estimated genetic correlations (r_g_) between models

	M2_BTR	M1_AFH	M2_AFH	M1_HL
M1_BTR	0.97	0.68	0.60	0.92
M2_BTR		0.69	0.72	0.68
M1_AFH			0.95	0.99
M2_AFH				0.99

Standard errors ranged from 0.002 to 0.02

Table 7 shows the descriptive statistics for the two groups of stallions per model: proven stallions with an EBV that can be published and young stallions (4–5 years old) with own performances but without offspring. Since the stallions that meet publication criteria had more own records and more offspring than the young stallions, their mean EBV accuracy was higher for all the models (0.82–0.83 vs 0.60–0.70) and they were within the same range. However, their mean EBV was lower than that for the young stallions for all the models. Nevertheless, a smaller mean number of offspring was found for M2_AFH compared to the two other models. Young stallions reached the highest mean EBV accuracy with M2_AFH (0.70) and the lowest with M2_BTR (0.60).

**Table 7:** Descriptive statistics for the two groups of stallions

	n	Mean EBV^a^	Mean EBV accuracy	Mean number of own records	Mean number of offspring
Stallions that meet publication criteria^b^					
M2_BTR	654	0.57	0.82	14.88	56.15
M2_AFH	841	1.06	0.83	11.69	46.91
M1_HL	766	1.21	0.83	0.26	50.20
Young stallions (4–5 years old)					
M2_BTR	1004	0.78	0.60	6.84	0.00
M2_AFH	1004	1.35	0.70	6.84	0.00
M1_HL	1004	1.49	0.69	1.00	0.00

^a^Mean EBV expressed in genetic standard deviations

^b^Proven stallions with EBV accuracy ≥ 0.70 and at least five offspring

**Fig. 2:**
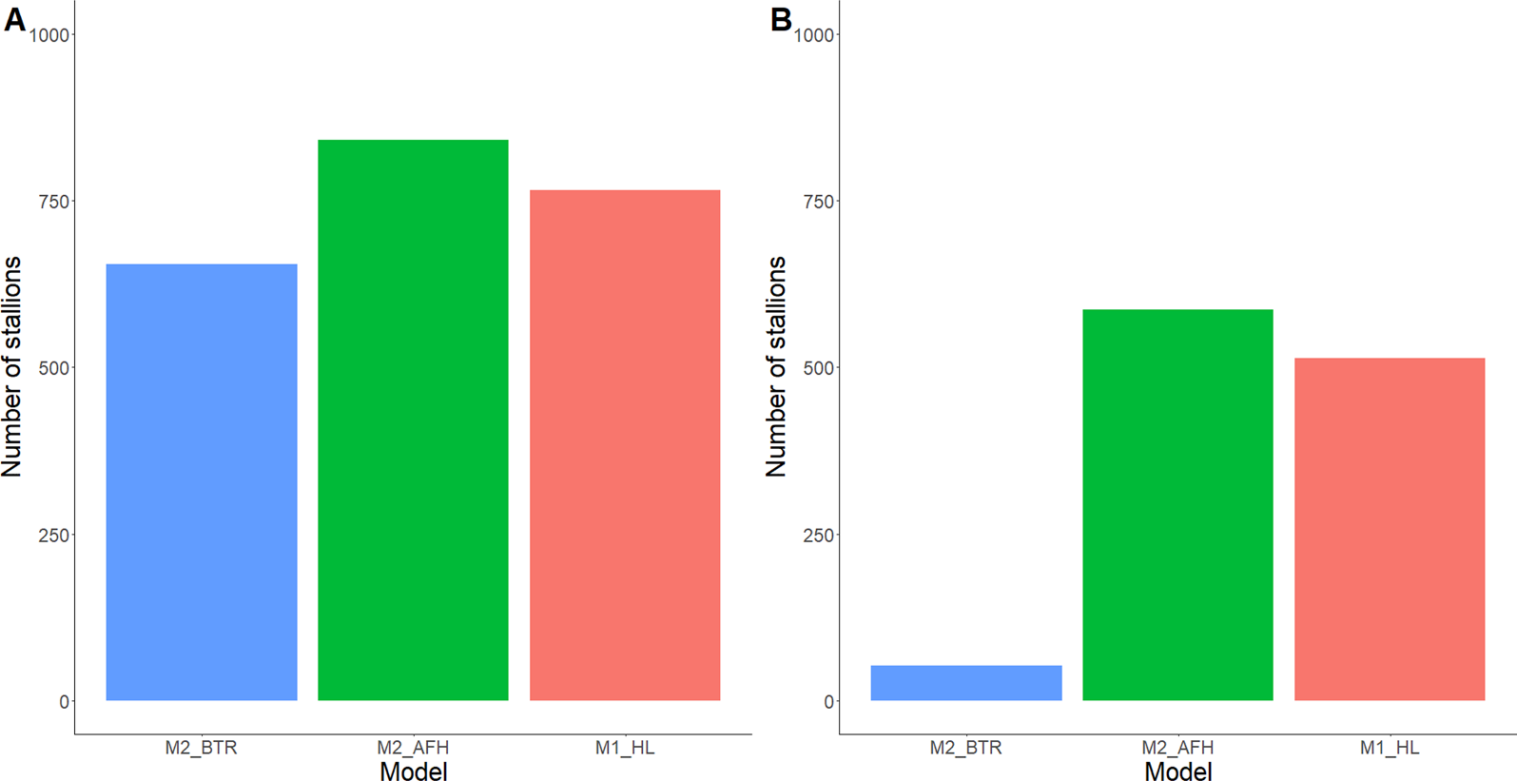
Number of stallions that reach an accuracy of estimated breeding value higher than 0.70: **a** stallions for which EBV can be published and **b** young stallions (4 to 5 years old). Publication criteria: having an accuracy of estimated breeding value ≥ 0.70 and at least five offspring with own performances

The number of animals that reach an EBV accuracy higher than 0.70 per group of stallions and per model is shown in Fig. 2. More stallions met the publication criteria (accuracy ≥ 0.70 and at least five offspring) for M2_AFH (n = 841, 8.1% of all stallions) compared to M2_BTR and M1_HL with 654 (6.3% of all stallions) and 766 stallions (7.4% of all stallions), respectively. The same held for the young stallions, with 586 young stallions reaching an EBV accuracy of 0.70 or more for M2_AFH compared to only 514 and 52 for M1_HL and M2_BTR, respectively).

**Fig. 3:**
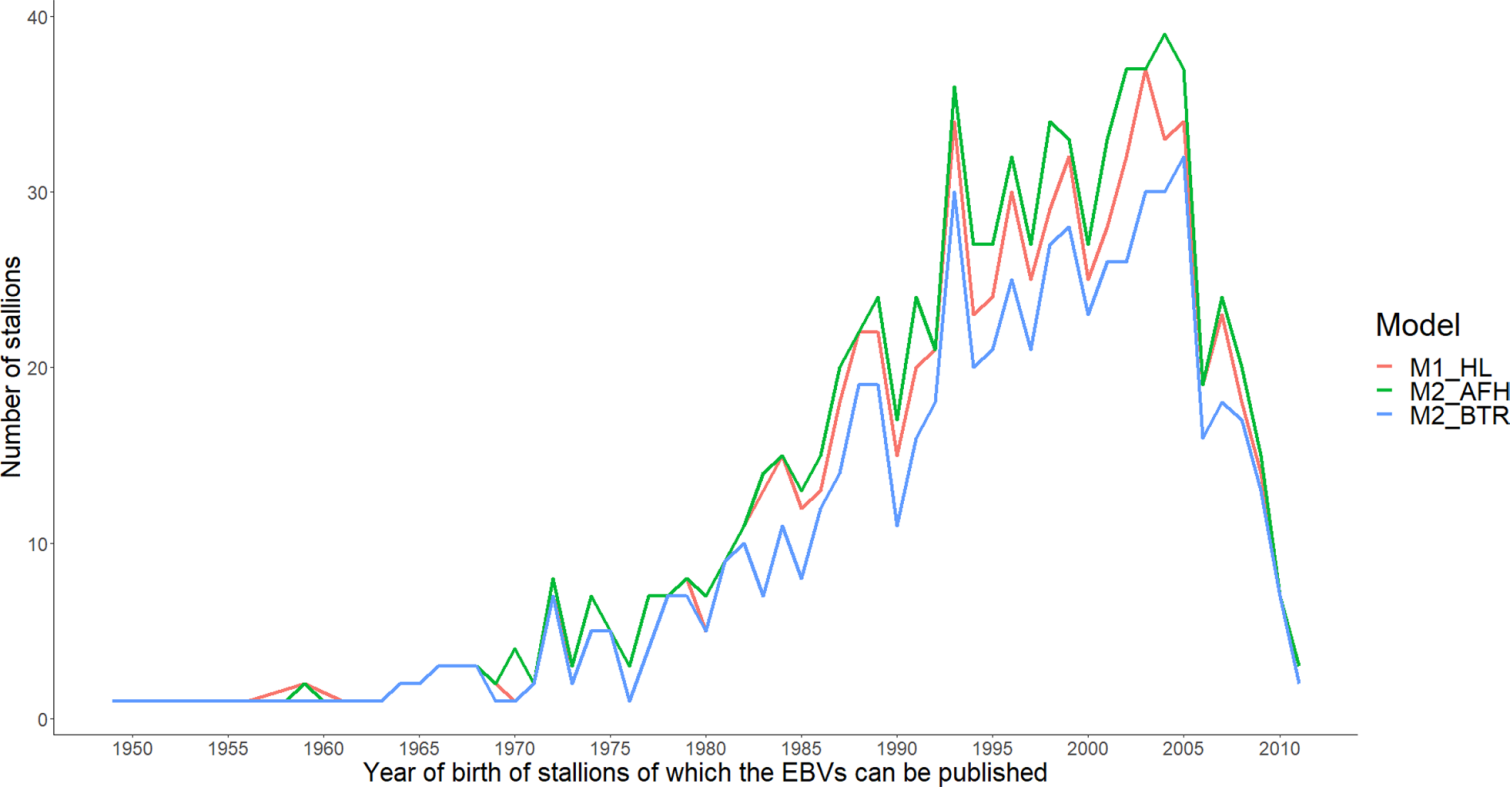
Number of stallions for which EBV can be published per birth year and per model. Publication criteria: having an accuracy of estimated breeding value ≥ 0.70 and at least five offspring with own performances

**Fig. 4:**
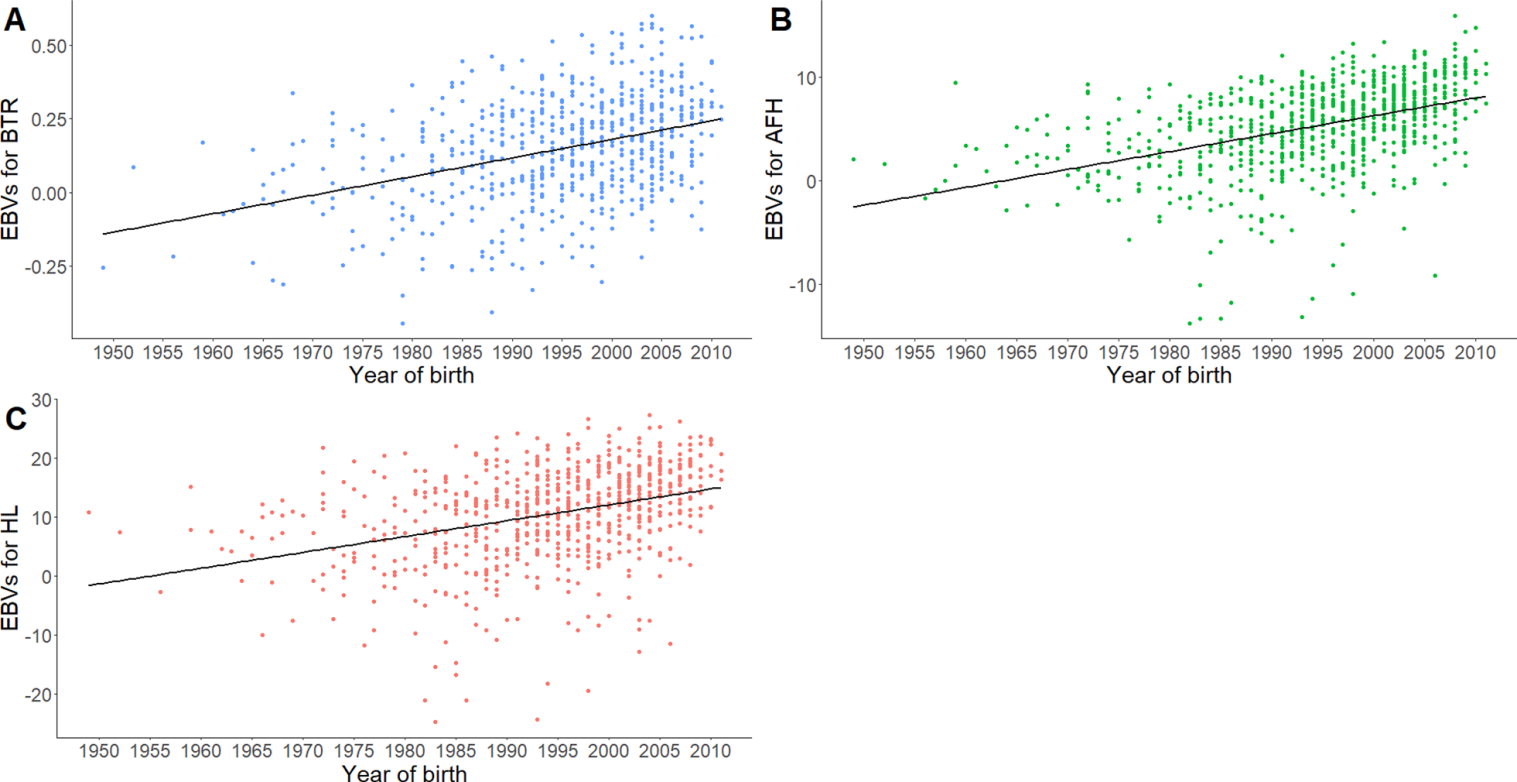
Relationship between year of birth of stallions and their EBV for the **a** M2_BTR, **b** M2_AFH, and **c** M1_HL models

The range of birth years of the approved stallions that meet publication criteria did not differ (1949 to 2011) between traits (Fig. 3). However, regarding year of birth, stallions tended to be younger for M2_AFH and M1_HL (Fig. 4).

Differences in Spearman rank correlations between EBV of approved stallions (that meet publication criteria) were also observed between models (Table 8), with values ranging from 0.69 to 0.88 and being highest (0.88) between M1_HL and M2_AFH and lowest (0.69) between M2_BTR and M1_HL and between M2_BTR and M2_AFH. Table 8 shows that the results obtained for the group of young stallions are similar to those of the approved stallions although their Spearman rank correlations were lower (r = 0.49–0.71 vs 0.69–0.88). The highest correlation was between M2_AFH and M1_HL and the lowest between M2_AFH and M2_BTR.

**Table 8:** Spearman rank correlations between EBV of approved stallions and young stallions

	Approved stallions		Young stallions	
	M2_AFH	M1_HL	M2_AFH	M1_HL
M2_BTR	0.69	0.69	0.49	0.53
M2_AFH		0.88		0.71

Publication criteria for approved stallions: accuracy of estimated breeding values ≥ 0.70 and at least five offspring with own performances per stallion

## Discussion

Ideally, genetic evaluations are based on EBV derived from informative phenotypes that can efficiently improve the breeding goal. In the case of show jumping, no single trait can be used to translate “success in competitions” into a measurable trait and several countries have developed their own custom phenotype since no gold standard has ever been adopted by the Warmblood studbooks. However, the same difficulty is encountered by any organization that aims at selecting for “success in competitions”, i.e. how can the intra-competition variability be differentiated from the inter-competition variability? Two different strategies are used in sport horse breeding for that purpose: (i) using a rank-based phenotype and correcting for the competition level by adding an event effect, or (ii) developing a phenotype based on (partly) arbitrary points that are assigned based on the ranking and competition level. Here, we studied BTR as a rank-based phenotype and developed a new phenotype AFH based on a non-arbitrary method that combines information from Blom-transformed rankings (ranking i.e. intra-competition variability) and fence height (competition difficulty i.e. inter-competition variability). In the genetic model used for BTR, an event effect was added to account for the inter-competition variability. However, in show jumping competitions, horses that compete in the same event(s) are not grouped at random i.e. they are pre-selected based on their level. Hence, adding an event effect is not sufficient because it cannot be estimated correctly and thus leads to biased EBV as shown in Ricard and Legarra [33]. Therefore, to account for intra- and inter-competition variability, it is better to use a phenotype such as AFH that measures directly ranking and competition difficulty from the data.

The use of traits based on elementary performances such as BTR and AFH allows the model to account for effects that are specific to each competition. Success in competitions is not only due to the horse’s jumping ability but also to the skills of the rider [34], thus accounting for a rider effect in the genetic model is relevant. Here, by comparing M1_BTR to M2_BTR and M1_AFH to M2_AFH, we showed that including a rider effect in the model improved the model fit according to the AIC values, which confirms previous studies in different equine disciplines: show jumping, dressage or trotting races [4, 15, 19, 23, 25]. Our estimated rider effect for AFH accounts for a larger proportion of the variance (0.51) than that cited in these papers, which may be due to a larger variation in riders’ skills and/or a larger number of riders in our data. In addition, by construction, we considered a zero correlation between rider effect and genetic effect. However, the comparison of M1_BTR with M2_BTR and of M1_AFH with M2_AFH showed that accounting for a rider effect decreased the residual variance and the sum of the genetic and permanent environment effects, which suggests that rider effect could be correlated with the genetic and permanent environment effects. Developing a model that includes an interaction between genetic and permanent environment effects could help dealing with this issue although convergence problems may occur. To alleviate this, we decided to filter the data by removing the records of horses that were ridden by only one rider (i.e. 6.1% of the total number of removed records). If these single rider performances are retained, distinguishing the rider effect from the horse effect would be more difficult and could lead to confounding effects as shown by Albertsdóttir et al*.* [35]. Contrary to traits based on elementary performances, a trait based on a summarized performance such as HL, does not allow accounting for differences between riders or only partly, when horses are ridden by several riders which is often the case in a horse career.

In this study, we evaluated two traits based on elementary performances (BTR and AFH) and one trait based on a summarized performance (HL). The heritability estimates for M2_AFH and M2_BTR (the best-fit models for AFH and BTR) are low but similar to those reported for traits related to jumping performance based on elementary performances (Table 1). For BTR, the heritability estimates range from 0.06 to 0.09 and are consistent with those reported for Belgian sport horses by Janssens et al. [3], which range from 0.02 to 0.10 although neither Z horses nor a rider effect were included (Table 1). M2_AFH presents a higher heritability estimate (0.12) than M2_BTR but it is still lower than the estimate obtained with M1_HL (0.39). With M1_HL the heritability estimate is a little higher than the range of estimates for the “highest level achieved in a career” traits found in the literature (from 0.10 to 0.36, see Table 1). The genetic correlations estimated between the models were high (0.60) to very high (0.99) (Table 6). The highest genetic correlations (r_g_ = 0.99) were found between M1_AFH and M1_HL and between M2_AFH and M1_HL. The fact that M1_BTR and M2_BTR differentiate the intra- from the inter-competition variability only because an event effect was added to account for the level of competitors may be the reason why they generally had lower genetic correlations with M1_AFH (r_g_ = 0.68 and 0.69), M2_AFH (r_g_ = 0.60 and 0.72) and M1_HL (r_g_ = 0.68 and 0.92). The Spearman rank correlations between the stallions’ EBV (approved and young stallions) (0.88 and 0.71 for M2_AFH and M1_HL and only 0.69 and 0.49 for M2_AFH and M2_BTR) also show that taking fence height and not only ranking into consideration results in a higher correlation between EBV for M2_AFH and M1_HL compared to the (lower) correlations with the EBV for M2_BTR.

In Belgium, two criteria are used to publish the EBV of stallions: having an EBV accuracy higher than 0.70 and at least five offspring with own performances. Published EBV are of great interest for breeders to help make mating choices. However, both the choice of the performance trait and the structure of the model have an impact on the list of stallions that meet the criteria. The use of M2_AFH would result in the largest number of stallions (841) (Fig. 3). Hence, this model returns more information to the breeders for making mating choices than M1_HL (766) or M2_BTR (654). The stallions for which EBV can be published based on M2_AFH and M1_HL also tend to be younger than the stallions based on M2_BTR, which indicates that the EBV of the stallions reach an accuracy of 0.70 at a younger age, hence with less own performances and/or fewer offspring with own performances. Moreover, more young stallions (4 to 5 years old with own performances but without offspring) reached an EBV accuracy of 0.70 with M2_AFH than with the other models (Fig. 2). Thus, our proposed AFH model will promote the use of younger stallions, which will impact generation interval and hence genetic progress.

Extending the current model by including a rider effect and using AFH presents substantial advantages to improve a selection program. As an axis for future research, including international competition records and not only national data could be interesting to assess the reproducibility of AFH and the added value of international data to the genetic evaluation.

## Conclusions

In this paper, we propose and evaluate a novel phenotype, i.e. adjusted fence height, for the genetic evaluation of show jumping performance in Warmblood horses. This phenotype combines the fence height used in a competition with the final ranking of horses in this competition, and thus differentiates the intra- from the inter-competition variability by using a novel non-arbitrary method that can be extended to any show jumping competition dataset. This new trait, used in a model that includes a rider effect is moderately correlated with the Blom-transformed ranking and strongly correlated with lifetime success (highest level achieved). In addition, the use of this phenotype results in more approved stallions with publishable EBV, more young males reaching an EBV accuracy of 0.70 and thus more information for the breeders. A revision of the current Belgian model for show jumping performance is therefore advocated.

## Data Availability

The datasets used being the property of the K.B.R.S.F., BWP and Z studbooks, their disclosure depends on them.

## References

[1] Koenen EPC, Aldridge LI, Philipsson J (2004). An overview of breeding objectives for warmblood sport horses. Livest Prod Sci.

[2] Bowling AT, Ruvinsky A (2000). The genetics of the horse.

[3] Janssens S, Geysen D, Vandepitte W. Genetic parameters for show jumping in Belgian sport horses. 48th Annual Meeting of the European Association for Animal Production: 25–28 August 1997; Vienna. 1997.

[4] Posta J, Komlósi I, Mihók S (2009). Breeding value estimation in the Hungarian Sport Horse population. Vet J.

[5] Posta J, Malovhr S, Mihók S, Komlósi I (2010). Random regression model estimation of genetic parameters for show-jumping results of Hungarian Sporthorses. J Anim Breed Genet.

[6] Foran MK, Reilly MP, Kelleher DL, Langan KW, Brophy PO. Genetic evaluation of show jumping horses in Ireland using Ranks in competition. 46th Annual Meeting of the European Association for Animal Production: 4–7 September 1995; Prague. 1995.

[7] Schubertová Z, Candrák J, Rolinec M (2016). Genetic evaluation of show jumping horses in the Slovak Republic. Ann Anim Sci.

[8] Rudiné Mezei A, Posta J, Mihók S (2013). Evaluation of Hungarian show-jumping results using different measurement variables. Acta Agrar Debr.

[9] Reilly M, Foran MK, Kelleher DL, Flanagan MJ, Brophy PO (1998). Estimation of genetic value of showjumping horses from the ranking of all performances in all competitions. J Anim Breed Genet.

[10] Aldridge LI, Kelleher DL, Reilly M, Brody POB (2000). Estimation of the genetic correlation between performances at different levels of show jumping competitions in Ireland. J Anim Breed Genet.

[11] Luehrs-Behnke H, Roehe R, Kalm E (2002). New integrated breeding evaluation method used for German warm-blooded horses. Acta Agrar Debr.

[12] Jaitner J, Reinhardt F. National genetic evaluation for horses in Germany. 54th Annual Meeting of the European Association for Animal Production: 31 August–3 September 2003; Rome. 2003.

[13] Tavernier A. Special problems in genetic evaluation of performance traits in horse. In Proceedings of the 5th World Congress on Genetics Applied to Livestock Production: 7–12 August 1994; Guelph. 1994.

[14] Novotná A, Bauer J, Vostrý L, Jiskrová I (2014). Single-trait and multi-trait prediction of breeding values for show-jumping performance of horses in the Czech Republic. Livest Sci.

[15] Bartolomé E, Menéndez-Buxadera A, Molina A, Valera M (2018). Plasticity effect of rider–horse interaction on genetic evaluations for Show Jumping discipline in sport horses. J Anim Breed Genet.

[16] Ducro BJ, Koenen EPC, van Tartwijk JMFM, Bovenhuis H (2007). Genetic relations of movement and free jumping traits with dressage and show-jumping performance in competition of Dutch Warmblood horses. Livest Sci.

[17] Welker V, Stock KF, Schöpke K, Swalve HH (2018). Genetic parameters of new comprehensive performance traits for dressage and show jumping competitions performance of German riding horses. Livest Sci.

[18] Huizinga HA, van der Meij GJ (1989). Estimated parameters of performance in jumping and dressage competition of the Dutch Warmblood horse. Livest Prod Sci.

[19] Rovere G, Ducro BJ, van Arendonk JAM, Norberg E, Madsen P (2016). Analysis of competition performance in dressage and show jumping of Dutch Warmblood horses. J Anim Breed Genet.

[20] Quinn-Brady KM, Hart D, Corbally A. The inclusion of international show jumping results in the genetic evaluation of Irish sport horses. 64th Annual Meeting of the European Association for Animal Production:26–30 August 2013; Nantes. 2013.

[21] Viklund A, Braam A, Näsholm A, Strandberg E, Philipsson J (2010). Genetic variation in competition traits at different ages and time periods and correlations with traits at field tests of 4-year-old Swedish Warmblood horses. Animal.

[22] Ricard A, DumontSaint Priest B, Chassier M, Sabbagh M, Danvy S (2020). Genetic consistency between gait analysis by accelerometry and evaluation scores at breeding shows for the selection of jumping competition horses. PLoS ONE.

[23] Kearsley CGS, Woolliams JA, Coffey MP, Brotherstone S (2008). Use of competition data for genetic evaluations of eventing horses in Britain: analysis of the dressage, showjumping and cross country phases of eventing competition. Livest Sci.

[24] Bartolomé E, Menéndez-Buxadera A, Valera M, Cervantes I, Molina A (2013). Genetic (co)variance components across age for Show Jumping performance as an estimation of phenotypic plasticity ability in Spanish horses. J Anim Breed Genet.

[25] Sánchez Guerrero MJ, Cervantes I, Valera M, Gutiérrez JP (2014). Modelling genetic evaluation for dressage in Pura Raza Español horses with focus on the rider effect. J Anim Breed Genet.

[26] Solé M, Bartolomé E, Sánchez MJ, Molina A, Valera M (2017). Predictability of adult Show Jumping ability from early information: alternative selection strategies in the Spanish Sport Horse population. Livest Sci.

[27] Chapard L, Buys N, Janssens S. Methodology to integrate pedigrees of two Belgian Warmblood studbooks and its importance for genetic evaluation. In Proceedings of the 12th World Congress on Genetics Applied to Livestock Production: 3–8 July 2022; Rotterdam. 2022.

[28] R Core Team. R: a language and environment for statistical computing. 2022. https://www.R-project.org/. Accessed 23 Jan 2023.

[29] Blom G (1958). Statistical estimates and transformed beta-variables.

[30] Misztal I, Tsuruta S, Lourenco DAL, Masuda Y, Aguilar I, Legarra A (2018). Manual for BLUPF90 family programs.

[31] Akaike H. Information theory and an extension of the maximum likelihood principle. In Proceedings of the 2nd International Symposium on Information Theory: 2–8 September 1973; Budapest. 1973.

[32] Dubois C, Manfredi E, Ricard A (2008). Optimization of breeding schemes for sport horses. Livest Sci.

[33] Ricard A, Legarra A (2010). Validation of models for analysis of ranks in horse breeding evaluation. Genet Sel Evol.

[34] McLean AN, McGreevy PD (2010). Ethical equitation: capping the price horses pay for human glory. J Vet Behav.

[35] Albertsdóttir E, Eriksson S, Näsholm A, Strandberg E, Arnason T (2007). Genetic analysis of competition data on Icelandic horses. Livest Sci.

